# Correlation of radiological features of white epidermoid cysts with histopathological findings

**DOI:** 10.1038/s41598-022-06167-x

**Published:** 2022-02-10

**Authors:** Dima Z. Jamjoom, Ali Alamer, Donatella Tampieri

**Affiliations:** 1 Department of Radiology and Medical Imaging, College of Medicine, King Saud University, P.O. Box 7805, Riyadh, 11472 Saudi Arabia; 2 Department of Radiology, College of Medicine, Qassim University, Buraidah, Saudi Arabia; 3 Kingston Health Sciences Centre, Queen’s University, Kingston, Canada

**Keywords:** Neurological disorders, Diseases of the nervous system

## Abstract

Epidermoid cysts are benign congenital extra-axial lesions commonly found in the posterior fossa. These lesions have a characteristic imaging appearance on computed tomography (CT) scan and magnetic resonance imaging (MRI), but occasionally they may exhibit atypical radiological features, showing unusual hyperintensity on T1-weighted images (T1WI). Currently, such atypical appearance is referred to as white epidermoid. We present the imaging features of 5 cases of white epidermoid cyst and discuss the possible underlying etiology of this unusual radiological appearance. We retrospectively searched our electronic radiology database from January 2005 to December 2015 for all intracranial epidermoid cysts, which were confirmed either by typical MRI appearance or histopathological examination. All white epidermoid cases were evaluated with non-enhanced CT scan and multisequential MRI. Histopathological correlation was carried out in four white epidermoid cases. A total of 61 patients with epidermoid cyst were found, of those 5 (8%) were considered white epidermoids. These consisted of 3 females and 2 males, ranging in age between 31–63 years (average age was 51.8 years). Three patients had lesions located in the posterior fossa. The 2 other patients had lesions in the suprasellar region, with extension to the right middle cranial fossa in one. All 5 lesions were hyperdense on CT scan and hyperintense on T1WI. One patient demonstrated evidence of transformation of a classic epidermoid to a white epidermoid after partial resection. Histopathologically, cholesterol clefts were seen in 3 epidermoid cysts, each which also showed microcalcifications, proteinaceous material or melanin. Hemorrhage was demonstrated in one additional lesion. White epidermoid cyst is an unusual intracranial lesion that should be considered when encountered with an extra-axial T1 hyperintense lesion. The cause of this hyperintensity is not clearly understood, but the presence of cholesterol, microcalcifications, proteinaceous content and rarely hemorrhage or melanin may be contributing factors.

## Introduction

Epidermoid cysts are benign congenital lesions that arise from retained ectodermal epithelium during neural tube closure between the third and fifth weeks of gestation^1^. They represent about 0.2–1.8% of all primary intracranial tumors^2^. Although typically congenital, epidermoid cysts can be acquired following surgery or trauma^3^. They have no gender predilection and typically occur between the ages of 20–60 years, with a peak incidence in the fourth decade^4^.

These lesions are slow-growing and typically present with non-specific signs and symptoms based on local mass effect on the adjacent neural and vascular structures^2^. Headache and seizures were reported as the most common presenting symptoms^5^; however, rapid onset of symptoms can occur secondary to hemorrhage, trauma, rupture with chemical meningitis and acute brain edema^6,7^.

Epidermoid cysts are extra-axial lesions, commonly found in the posterior fossa, with the cerebellopontine angle cistern being the most common location^1^. On imaging they classically demonstrate hypodensity on CT scan, hypointensity on T1WI, and hyperintensity on T2-weighted images (T2WI) almost similar to cerebrospinal fluid (CSF), with characteristic diffusion restriction on diffusion weighted imaging (DWI)^8–10^. Occasionally, they may exhibit atypical radiological features, showing hyperdensity on CT scan and spontaneous hyperintensity on T1WI. Such atypical imaging appearance is currently described as white epidermoid cyst.

In this series, we present the imaging features of 5 cases of atypical “white” epidermoid cyst and discuss the possible underlying etiology of this unusual radiological appearance. The physiochemical basis of such atypical appearance is reviewed based on the histopathological results and the relevant data in the literature. Furthermore, the possible transformation of classic epidermoid into white epidermoid cyst was discussed, which has not been previously reported in the literature.

## Material and methods

After institutional review board approval was obtained from McGill University Health Centre (MUHC) Research Ethics Board, we retrospectively reviewed the electronic radiology database from January 2005 to December 2015 for all intracranial epidermoid cysts. All study methods were performed in accordance with the institution’s guidelines and regulations. Informed consent was waived by the MUHC research ethics board due to the retrospective nature of the study. Cases were confirmed by typical imaging features of epidermoid cysts and/or histopathological examination. Only cases with epidermoid cysts demonstrating high signal intensity on T1WI were included.

Imaging of these patients included both brain CT scan and MRI. CT scan images were acquired in the axial plane using standard imaging protocol without administration of IV contrast medium, followed by sagittal and coronal reconstructions. MRI was performed using Philips Ingenia 1.5 T or GE signa 1.5 T with an 8-channel head coil. Routine brain protocol was acquired which included 3D spin-echo (SE) T1WI with imaging parameters of 400 ms (TR) and 8.8 ms (TE), section thickness of 1.24 mm/0.62 mm, a matrix of 200 × 200 pixels, and FOV of 250 × 250 mm. Axial T2WI was also acquired with 5145 ms (TR) and 100 ms (TE), section thickness of 5 mm/6 mm, a matrix of 408 × 317 pixels, and FOV of 245 × 245 mm. The protocol also included 3-dimensional fluid-attenuated inversion recovery (FLAIR), DWI and susceptibility weighted sequences (SWI). Post contrast imaging was acquired in all patients in the axial and coronal planes after intravenous administration of 1 cm^3^/10 kg Gadovist 1.0 (Gadobutrol) at rate of 2.0 mL/s using a power injector. In one patient, the lesion was located in the sellar/suprasellar region and dedicated imaging of the sella turcica was obtained, which included sagittal and coronal T1WI, coronal T2WI and post contrast images in sagittal and coronal planes.

All studies were reviewed by 1 senior neuroradiologist and 2 neuroradiology fellows. Lesion location and CT and MRI characteristics were documented, which included CT density, T1 and T2WI signal intensity, DWI and SWI features and post contrast enhancement. Histopathological correlation was carried out when available.

## Results

A total of 61 epidermoid cysts were found in our radiological database. The patients included 32 females and 29 males, ranging in age from 13 to 87 years (average age was 54 years). Of these cases, 5 patients had white epidermoid cysts, demonstrating high signal intensity on T1WI. This represented 8% of all intracranial epidermoid cysts in our institution. They included 3 women and 2 men, ranging in age from 31 to 63 years (average age was 51.8 years). Three of these patients presented with headache, and one patient with hypopituitarism and vision loss due to involvement of the sellar region. Another patient was known to have a previous partially resected classic epidermoid cyst in the suprasellar region extending to the right middle and posterior cranial fossae, and presented for resection of an enlarging residual lesion.

**Figure 1:**
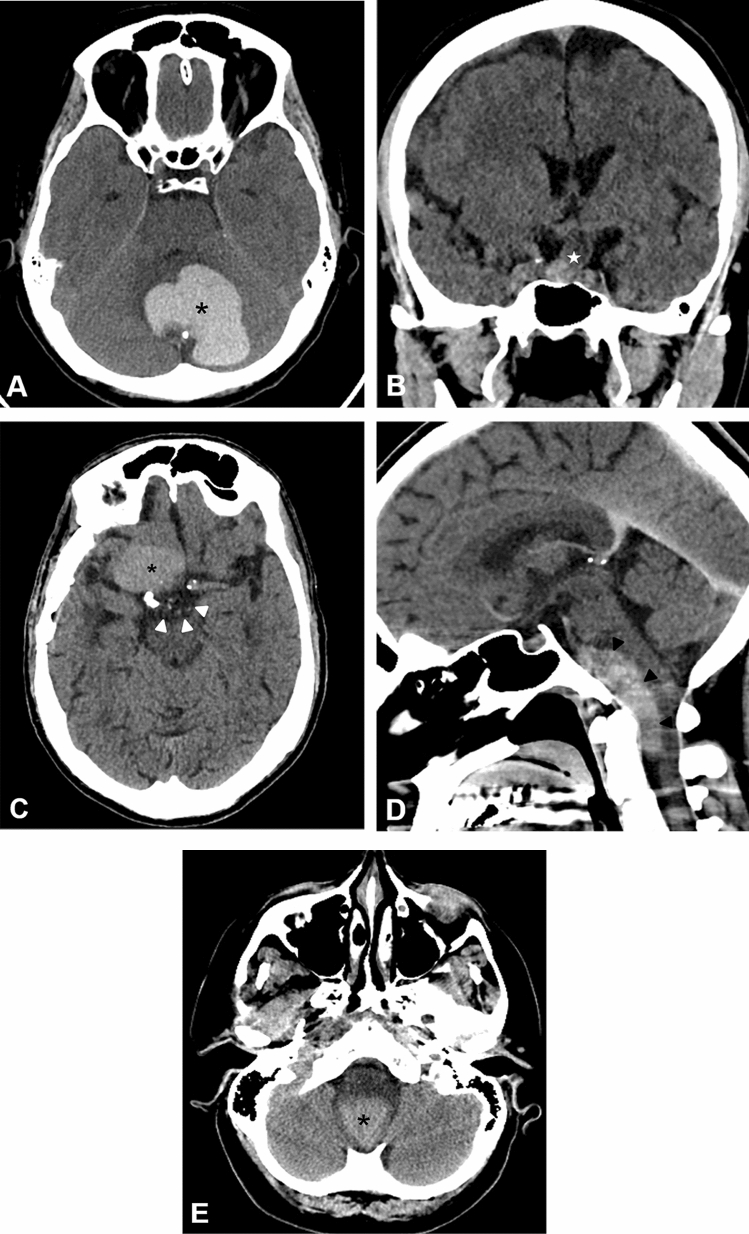
Plain CT scan images of the brain of the 5 patients with white epidermoid cyst. The lesions exhibit variable spontaneous hyperdensity. Patient 1 (**A**) has a lesion in the posterior fossa in the retrocerebellar region (black asterisk) with a small focus of peripheral calcification. Patient 2 (**B**) presents with a sellar/suprasellar hyperdense lesions (white star). Patient 3 (**C**) has an epidermoid cyst with 2 components: classic epidermoid (white arrows) in the suprasellar region and white epidermoid (black asterisk) in the right middle cranial fossa, which developed after partial resection. Patient 4 (**D**) presents with a lesion located in the posterior fossa anteriorly, extending to the craniocervical junction (black arrowheads). Patient 5 (**E**) has a foramen magnum epidermoid cyst with extension to the spinal canal (black asterisk).

**Figure 2:**
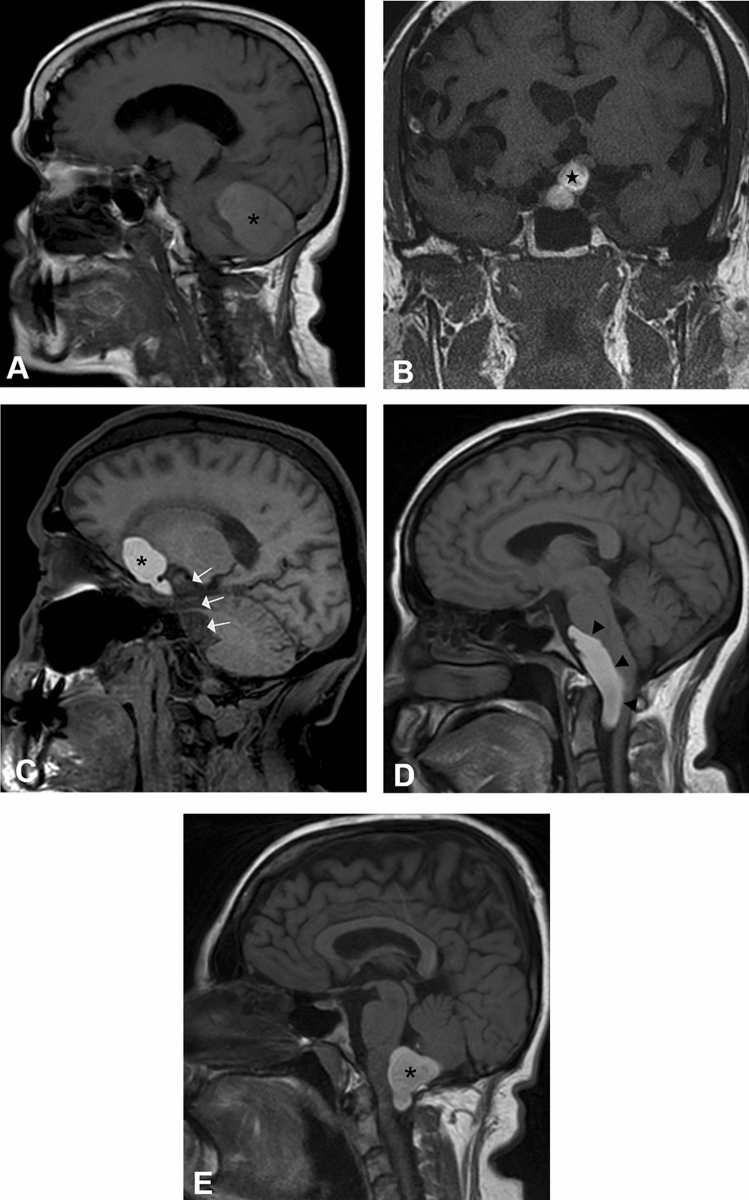
Non-enhanced T1-weighted MR images of the brain demonstrate spontaneously hyperintense white epidermoid cysts. Patients 1, 4 and 5 (**A**,**D**,**E**) have lesions in the posterior fossa, with extension to spinal canal in patients 4 (black arrowheads) and 5 (black asterisk). Patient 2 (**B**) presents with a sellar/suprasellar lesion (black star). The lesion in patient 3 (**C**) has 2 components: classic epidermoid posteriorly (white arrows) and white epidermoid anteriorly (black asterisk).

**Figure 3:**
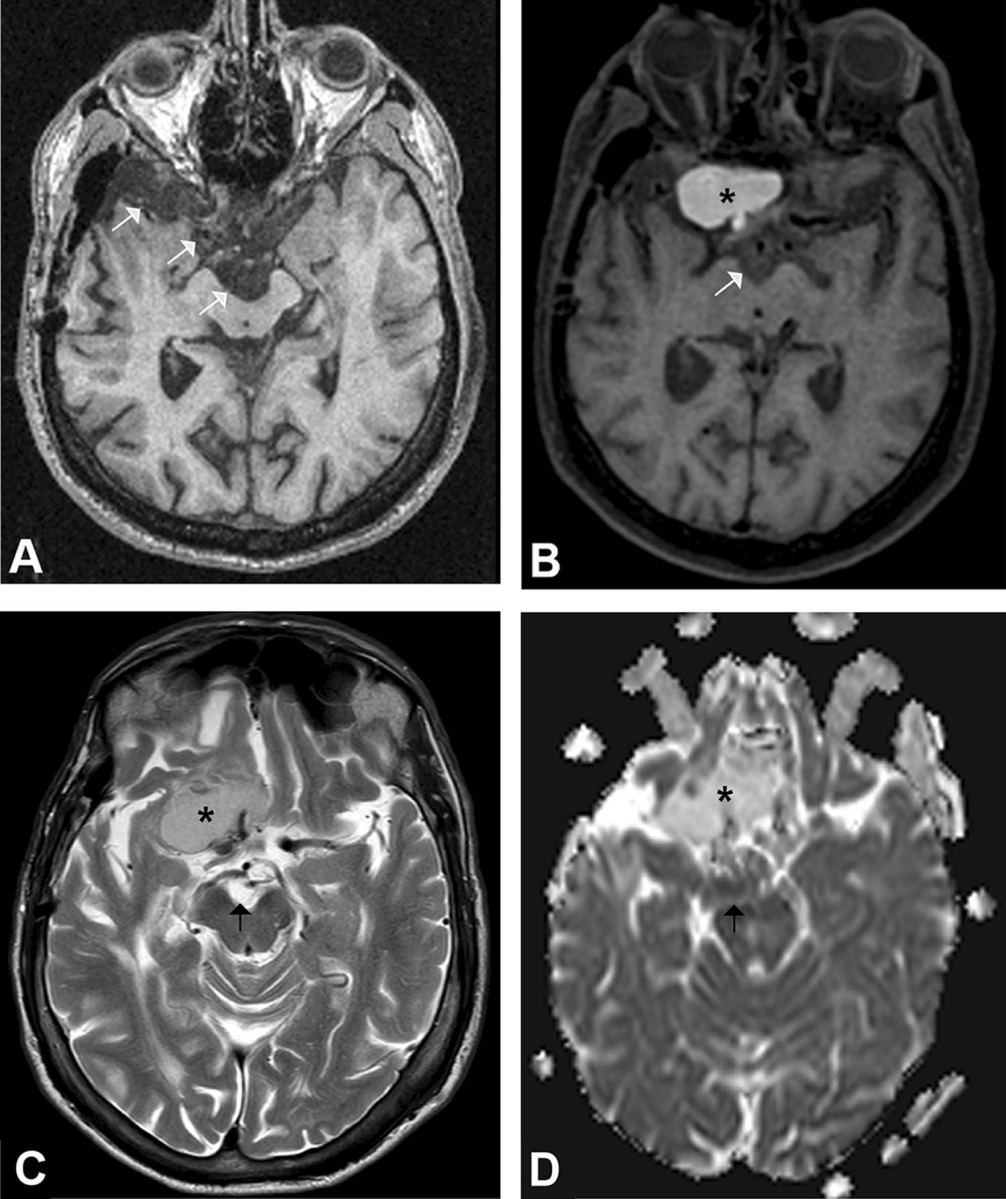
(**A**,**B**) Axial nonenhanced T1-weighted MRI in patient 3, which shows evidence of transformation of a residual classic epidermoid cyst in the suprasellar cistern and right middle cranial fossa (white arrows in **A**) to a white epidermoid (black asterisk in **B**). The interpeduncular component retained its classis epidermoid features (white arrow in **B**). The 2 images are taken 10 years apart. (**C**,**D**) Axial T2WI and ADC map demonstrating different signal intensities of the white epidermoid component (black asterisk) and classic epidermoid cyst (black arrow). The white epidermoid cyst shows intermediate signal intensity on T2WI and high signal intensity on the ADC map, indicating lack of diffusion restriction, in contrast to the classic epidermoid, which demonstrates high T2 signal intensity and diffusion restriction.

All lesions were lobulated and extra-axial in location. Three of the lesions were primarily located in the posterior fossa, with extension to the craniocervical junction in 2 cases. Two lesions were in the suprasellar region, one of them extending to the right middle and posterior cranial fossae. On CT scan, all lesions showed variable degrees of spontaneous hyperdensity, ranging from 49.14 to 72.67 Hounsfield Units (HU) with an average of 55 HU (Fig. 1). A small focus of gross peripheral calcification was noted in only one posterior fossa white epidermoid cyst. All lesions were spontaneously homogeneously hyperintense on T1WI (Fig. 2), with variable signal intensity on T2WI, ranging from isointense in one case to hypointense in 4 cases, 3 of which were markedly hypointense. The spontaneously T1 hyperintense signal of the lesions did not demonstrate diffusion restriction on DWI, susceptibility artifact on SWI nor enhancement on the post contrast images. One of the patients showed evidence of transformation of a partially resected classic epidermoid cyst in the suprasellar cistern and right middle cranial fossa to a white epidermoid cyst. The patient had a component in the posterior fossa that showed classic features (Fig. 3).

Four of the 5 white epidermoid cysts underwent surgical resection. The histopathological examination showed a fibrous wall lined by keratinizing squamous epithelium and enucleated squamous cells without skin appendages or hair follicles, confirming the diagnosis of epidermoid cyst in all resected cases. Cholesterol clefts were present on microscopy in 3 white epidermoid cysts (75%), along with microcalcifications and ossification, proteinaceous material and melanin in 1 case each (25%). Hemorrhage was present in 1 case (25%). These microscopic findings may possibly explain the cause of the spontaneous T1 hyperintensity in our cases. One case with cholesterol clefts and melanin also demonstrated granulation tissue containing multinucleated giant cells and lymphocytic infiltrates suggestive of a foreign body-like reaction. The fifth patient with the newly developed white epidermoid cyst in a formerly classic epidermoid did not undergo resection of the atypical T1 hyperintense component. The new atypical component in this case was diagnosed as white epidermoid cyst based on CT hyperdensity and T1 hyperintensity, in addition to the histopathological confirmation of its classical component. Furthermore, no diffusion restriction, susceptibility on SWI, or contrast enhancement was noted in the atypical component.

Patient demographics, clinical presentation, lesion imaging features and histopathological correlation are summarized in Table 1.

**Table 1:** Patient demographics, clinical presentation, lesion imaging features and histopathological correlation.

No.	Age	Gender	History	Location	CT scan	T1WI	T2WI	Histopathology
1	63	Female	Headache, ataxia and dysmetria	Posterior fossa (retrocerebellar)	Hyperdense (72.67 HU)	Hyperintense	Markedly hypointense	Epidermoid cyst with microcalcifications and cholesterol clefts
2	59	Male	Hypopituitarism and progressive vision loss	Sellar/suprasellar	Hyperdense (54.15 HU)	Hyperintense	Markedly hypointense	Epidermoid cyst with hemorrhage
3	63	Male	Previous resection of a classic epidermoid cyst	Suprasellar/right middle cranial fossa with extension to the posterior fossa	Hyperdense (49.14 HU). The posterior fossa component is hypodense	Hyperintense. The posterior fossa component is hypointense	Isointense. The posterior fossa component is hyperintense	Classic epidermoid cyst. The white epidermoid component was not resected
4	31	Female	Headache	Posterior fossa with extension to the craniocervical junction	Hyperdense (49.96 HU)	Hyperintense	Hypointense	Epidermoid with melanin, cholesterol clefts, multinucleated giant cells and lymphocytic infiltrates
5	43	Female	Headache	Posterior fossa (foramen magnum) with caudal extension into the spinal canal	Hyperdense (49.31 HU)	Hyperintense	Markedly hypointense	Epidermoid with cholesterol clefts and proteinaceous material

## Discussion

Epidermoid cysts are benign congenital lesions that are formed from retained ectodermal epithelium^11^. The majority of epidermoid cysts are found in the cerebellopontine angle cistern, as we found in our series, followed by the prepontine, suprasellar and parasellar regions^1^. On gross pathology, epidermoid cysts are well-circumscribed lesions with a nodular surface and shiny “mother of pearl” appearance, from which the term “pearly tumors” is derived^2,9^. Calcification may sometimes be present^2,8^, as seen in one of our cases. They are lined by stratified squamous epithelium with cilia which undergoes progressive desquamation and keratin breakdown, resulting in lesion growth over time. These cysts are filled with soft, waxy or flaky material rich in debris, keratin, water and cholesterol crystals^11^. The typical imaging appearance is that of a well-defined lobulated mass which is hypodense on CT scan, hypointense on T1-weighted MRI, hyperintense on T2-weighted and DWI^12,13^. The hyperintense signal on DWI corresponds to hypointense signal on apparent diffusion coefficient (ADC) maps^9,13^. Such diffusion restriction indicates the solid nature of the lesion and restricted water diffusion to differentiate epidermoid cyst from other CSF-filled cysts such as arachnoid cyst^12,13^. The diffusion restriction in epidermoid cyst can be explained by the precise spatial organization of the lesion and tendency to grow in multiple epithelial layers^13^.

Occasionally, epidermoid cysts may appear hyperdense on CT scan and hyperintense on T1-weighted MRI. The T2 signal intensity of these atypical epidermoid cysts is variable, ranging from hyperintense to profoundly hypointense^8,14–18^, as noted in 3 cases in our series. Different terms have been used to describe such atypical radiological appearance. In 1977, it was originally described by Braun et al. as “dense epidermoid cyst” based on its hyperdense CT appearance^4^. This hyperdensity on CT scan did not necessarily correlate with hyperintensity on T1WI^9^. Currently, atypical epidermoid cysts are defined as those with spontaneous hyperintensity on T1WI and described as “white epidermoid cysts”. Surprisingly, white epidermoid cysts demonstrate lack of diffusion restriction in all our cases similar to prior reports compared to the classical epidermoid cysts^6,16,18^. Bohara et al. attributed the hypointense signal on DWI to xanthochromic fluid content of the cyst as a result of microbleeding or degenerative changes^16^. Li et al. explained such finding due to the liquid nature of the lesion^6^.

Although the exact number of published cases of white epidermoid cyst cannot be estimated precisely, not many cases have been reported in the literature. These lesions constitute about 3% of all epidermoid cysts^6^. The higher percentage (8%) of white epidermoid cysts in our study may be explained by the relatively small sample size. In our series, white epidermoid cysts showed a slight female predilection, as noted in previous series; however, the average age in our population was higher than reported in the literature (51.8 years compared with 37.5 years)^6^. We found that white epidermoid cysts occurred at a slightly younger average age (51.8 years) compared to the classic type (54 years). White epidermoid cysts are typically located in the sellar/suprasellar region, middle and posterior cranial fossae^8^, and this was also observed in our series. Intra-axial location of white epidermoid cysts is extremely rare and has been previously reported in the frontal lobe and brainstem^19,20^.

On gross inspection, atypical epidermoids are cystic in nature and contain fluid of various colors^21^. Although many authors have tried to explain the physiochemical basis of the unusual imaging features of these atypical “white” epidermoid cysts, the underlying etiology remains not clearly understood. Braun et al. attributed the high attenuation of dense epidermoid cysts on CT scan to diffuse calcifications and saponification of internal debris^4^. Other authors believe the dense aspect of the epidermoid is related to diffuse calcifications and ossifications of the thickened capsule and high protein content of the cystic fluid due to a proliferative and exudative defense reaction secondary to minor leak of the lepidic material through the cyst capsule^14,21^, which probably explains the T1 hyperintensity in only one of our cases. Following leakage of the cyst content, an immediate chemical inflammatory reaction occurs with fibroblast proliferation, which may lead to aseptic focal necrosis of the epithelial lining and liquefaction. With recurrent minor leaks, the cyst fluid becomes rich in protein and dystrophic calcium deposition can be seen^21^. Furthermore, such recurrent processes may induce perilesional vascular granulation tissues, which can compress blood vessels around the cyst, possibly resulting in spontaneous hemorrhage^6^. Epidermoid cysts can be associated with recurrent meningitis secondary to spontaneous leak, characterized by polymorphonuclear pleocytosis in cerebrospinal fluid^22^. This pleocytosis may also exist in the cyst fluid^6^.

Based on chemical analysis of the cyst fluid, Timmer et al. concluded that the imaging appearance of white epidermoids was related to high total protein concentration and the large albumin fraction. The concentrations of calcium and iron in the fluid were too low to account for the high density on CT scan^8^. According to quantitative analysis of cyst fluid in craniopharyngiomas, a protein level greater than or equal to 90 g/L can increase the signal intensity of fluid on T1WI. As protein levels increase, there is a gradual increase followed by a decrease in T1 signal intensity of cyst fluid^23^. The very high protein concentrations, in addition to high viscosity, may also explain the hypointense signal on T2WI^8^. Such T2 shortening of spontaneous T1 hyperintense cyst has been originally described as the “shading sign” in endometriotic cysts^24^. The “shading sign” was later also used in white epidermoid cysts^25^.

As noted in one of our cases, intracystic hemorrhage was believed to be the cause of high T1 signal intensity in some atypical epidermoid cysts because of the paramagnetic effect of heme iron (Fe^3^) in methemoglobin and other hemoglobin degradation products, although this is a rare occurrence due to the avascular nature of epidermoid cysts^26^. In 1981 Hasegawa et al. reported a case of an anterior cranial fossa dense epidermoid cyst on CT scan mimicking a meningioma and found the lesion to be hemorrhagic. They explained the cause of internal hemorrhage as a result of a small fibrous nodule containing numerous vessels and hemosiderin^27^. Furthermore, hemorrhage was also documented in an epidermoid cyst following head injury^28^.

A peculiar finding on pathological examination in one of our cases in this series is the presence of melanin deposits, which has been previously reported in only one case as a cause of spontaneous T1 hyperintensity in epidermoid cysts^29^. Along with melanin, numerous cholesterol clefts, multinucleated giant cells and lymphocytic infiltrates were present, which support the theory of an underlying minor leak causing an exudative defense reaction. Three of our cases had cholesterol clefts, which may possibly be the cause of T1 hyperintensity. Although chemical analysis was not performed in any of our cases, it has been reported that cholesterol in concentrations less than about 7 mmol/L would not account for T1 hyperintensity of cyst fluid^8,23^.

A patient in our series demonstrated usual progression of a partially resected epidermoid cyst in the suprasellar cistern and the right middle cranial fossa with classic features into a white epidermoid showing progressive T1 hyperintensity over a period of 10 years; a finding that has never been reported in the literature. We believe some intracystic bleed along with exudative defense reaction and possible recurrent minor infection and inflammation resulting in high protein content and polymorphonuclear pleocytosis may be causative factors of the spontaneous T1 hyperintense component, although this needs to be further validated by histopathological examination and chemical analysis. These changes may be the result of a chemical inflammatory response secondary to recurrent leakage of cyst content as previous reported by Li et al.^6^.

As with classic epidermoid cysts, the treatment of choice of white epidermoid cysts is microsurgical evacuation of the cyst content, in addition to radical resection of the capsule to avoid recurrence and the risk of chemical meningitis^30^. Li et al. reported higher incidence of capsular invasion with white epidermoid cysts as a result of the associated inflammatory reaction which may contribute to subtotal resection^6^. It was also found that white epidermoid cysts have a higher risk of leakage and subsequent chemical meningitis compared to classic epidermoid and even dermoid cysts^31^; therefore, making the diagnosis preoperatively may have implications in surgical planning and resection.

The differential diagnosis of white epidermoid cysts includes all lesions demonstrating hyperdensity on CT scan and/or hyperintensity on TIWI such as lesions with fat, proteinaceous, melanotic and hemorrhagic components^32^. These lesions include, but are not limited to dermoid cyst, neuroenteric cyst, craniopharyngioma, melanoma and cavernoma. Dermoid cysts usually present in younger patients and are midline in location, while epidermoid cyst is located laterally^33,34^. Furthermore, the presence of fat within the lesion is suggestive of dermoid cyst. Epidermoid cysts can be mistaken for neuroenteric cysts on CT scan^35^; however, neuroenteric cysts are typically hyperintense on T2WI as opposed to the usual T2 hypointensity of white epidermoid cysts^36^. The lack of contrast enhancement and calcifications excludes other neoplastic processes, such as craniopharyngioma and melanoma. Furthermore, the exceedingly rare intra-axial white epidermoid cysts can be initially diagnosed as cavernoma and glioma^20^.

We acknowledge some limitations of our study. The sample size was limited as a result of the rarity of white epidermoid cysts, which can be overcome by recruiting more centers. Furthermore, chemical analysis of the cysts was not performed in any of our patients, which can greatly aid in understanding the physiochemical basis of the unusual imaging appearance, and in particular the cause of the transformation. Finally, no routine post-operative imaging was obtained to assess for residual or recurrence.

White epidermoid cysts are unusual intracranial lesions that should be considered when encountered with an extra-axial spontaneously T1 hyperintense lesion. The cause of this hyperintensity is not clearly understood, and different authors attributed it to different factors. We believe as previously reported, that the presence of proteinaceous content, cholesterol, microcalcifications, and rarely hemorrhage or melanin may be contributing factors. Further studies with chemical analysis of the cyst fluid may be helpful to better understand the underlying etiology of this unusual imaging appearance.
